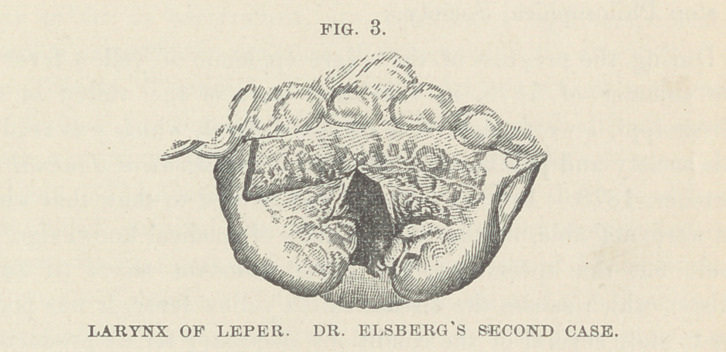# A Clinical Lecture on Tubercular Leprosy

**Published:** 1879-12

**Authors:** James Nevins Hyde


THE
CHICAGO MEDICAL
JOURNAL & EXAMINER.

VOL. XXXIX.—DECEMBER, 1879.—NO. 6.

[In these pages dimension and weight are expressed in terms of the Metric System, and temperature in degrees of the Centigrade Scale.]

### Original Lectures.

ARTICLE I.

A CLINICAL LECTURE ON TUBERCULAR LEPROSY, delivered at the Dermatological and Venereal Clinic, Rush Medical College, Sept. 29, 1878, by JAMES NEVINS HYDE. Reported by Mr. Philip Leach.

GENTLEMEN :— For the patient whom you see before you, we are indebted to Dr. M. Youngstedt, of this city, who is present and gives us the following history of the case: Peter Nanzen, age, 43, years; native of Angermanland. In this part of Sweden, there are men and women affected with a disease somewhat similar to that from which he now suffers, of whose nature he is quite ignorant. He remembers that the medical men of his native town were required to report to the proper authorities, all cases of this peculiar malady which came under their observation.

With regard to his own family history, he informs us that his father had some species of sore on the leg, the result of an injury, but that this parent survived till his 76th year. Our patient does not believe that the former was affected with the disorder from which he himself is suffering. His mother was always healthy, but one of his brothers when ten years of age, had a small brownish "spot" appear upon the surface of his body, which degenerated into an ulcer, and which subsequently required his removal to a hospital, where he died in his 22d year.

A second brother came to America, and died after exhibiting symptoms of similar character.

He states that he was married to a wife of his own nationality while residing in Sweden, and that they have had seven children born to them. The record of these is as follows:

1. Boy. Died in his 2d year, of "heart disease."
2. Boy. Died, 6th year, of "pneumonia."
3. Boy. Died, 3d month, of "croup."
4. Boy. Died, 2d year, of "croup."

These children were all born in Sweden. The following were born in America:

5. Girl. Living, in good health.
6. Girl. Living in her parents' home. She has from ten to fifteen "lumps" on her legs, similar to those which we shall observe in the case of the father. I think it proper for reasons which I will furnish later, to give her name in full, Lydia Margherita Nanzen.
7. Girl. Living in good health.

Our patient and his wife came to this country in October, 1868, eleven years ago. They first visited Omaha, but, remaining there for a short time only, settled finally upon a farm in Wahoo, Sanders county, Nebraska, where they are at present, comfortably and happily engaged in the healthful occupations of farm life.

As to his personal history, our patient informs us that he has never had a venereal disease of any kind, and that, with the single exception of a temporary illness accompanied by severe headache, with which he was seized at the time of his first coming to this country, he has always enjoyed excellent health up to the onset of his present disorder.

About five years ago, without the previous occurrence of chills, malaise or perverted sensation, he discovered a small "lump" on the superior and internal face of the right thigh, which still persists. Others followed in its near vicinity. Later, similar "lumps" appeared upon the forehead and arms. Meantime, his voice became husky, and he noticed that "lumps" similar to those upon the surface of the skin, could be seen in the throat. During the time which has elapsed since the appearance of the tubercles, he has gradually become weak. He is unable to work as much as formerly, and even locomotion is at times prostrating. His appetite is variable; his dejections, normal; the acuteness of the sense of taste and smell, somewhat diminished. He has correspondingly lost in weight. There has been an occasional feeling of numbness in the left leg with swelling of the hands, but never at any time, pricking or tingling sensations in any part of the body.

He has been under the charge of several "physicians," who have treated him for "scrofula." Under the advice of one of these gentlemen, he took the iodide of potassium for one year and a half, with scarcely appreciable effect. For the last two years, however, he informs me that he has been incapable of effecting sexual intercourse. Another "physician" removed one of the "lumps" on the forehead with the knife, but the little tumor did not fail to re-appear in precisely the same locality. Still another determined to destroy the lesions with caustic, but had no better success than had those who had experimented before him. I suppose that it is hardly necessary to state that these men all promised him relief. He is inclined to believe that when he was under the influence of arsenic, he was temporarily improved.

Let us now examine his condition for ourselves. You all see that he is a well-developed man, with an abundance of light brown hair on the scalp. Upon the surface of his forehead, we can count as many as 19 very firm, prominent, painless tubercles, varying in size from that of a pea to a walnut. There are, besides, several minute nodules in the skin of the part, which are, without question, undeveloped lesions of the same character. The largest of these is situated immediately over and partially involves the left eyebrow. It measures exactly 6 by 12 millimeters. In its center we can observe a cup-shaped shallow depression, where there has been absorption of the hyperplastic material from which the tubercle was developed. This was, possibly, an arrested process of ulceration. All the other tubercles are roundish, smooth, glistening or glabrous, brownish-red in color, and seated on what appears to be an entirely normal integument. There is no peripheral telangiectasis, but I can discover small enlarged vessels in a few of the new growths.

There is almost complete alopecia of the brows and lids, a few hairy filaments only can be seen on the upper lids. There are none whatever upon the lower lids. These few remaining filaments are short, deficient in pigment and easily removed.

In places there is slight circumscribed nodulation of the lobes of the ears, although there is here no distinct development of tubercles.

The teeth and gums are in a sound condition. Extending, however, from the middle of the hard through the soft palate, a tongue-shaped patch of split-pea-sized tubercles, its point ending in the thickened and tuberculated uvula, displays multiple lesions. These have a grayish-white depressed summit, due, as we learn, to the fact that caustic has been applied to each by the "physicians," to whose performances we have already alluded. When our patient speaks, you notice his peculiarly gruff, hoarse and discordant voice, from which it requires but little skill to conclude that the larynx is involved in the same pathological process. As we have not the requisite facilities in this room, I shall examine his vocal organs with the laryngoscope at the conclusion of this lecture, and will report to you the result.

There is a moderate degree of dyschromia to be perceived over the general surface of this man's body. Between the scapulæ, are distinct bronze-colored streaks, less marked upon the abdominal surface. The skin of the face also, is one or two shades darker than that noted usually in men of his complexion and nationality.

Extensive inguinal adenopathy exists on both sides, though the post-cervical, epitrochlear and other glands are not involved. The glands of the groins vary in size from pigeon's to hen's egg, and are painlessly involved, the overlying integument remaining unchanged.

Almost immediately over the right olecranon process, is a singular lesion. which differs markedly from the tubercles we have seen elsewhere. It is a smooth, yellowish-red plâque, looking something like a condyloma, but is very firm to the touch and quite solid in structure. It is evidently an infiltration of the entire thickness of the skin, in a circular patch measuring 5 centimeters in each diameter, and raised to the extent of about one-half a centimeter. I think that its resemblance to a slice of bacon in the skin would occur to you without suggestion of mine. The patch is generally smooth, though there are a few firmly attached scales in one portion. The history of this lesion differs from that of the others on the same arm. He states that he first had a "blister" develop in its site, which became afterward an open sore, lasting for six months. Even after this healed, it reopened and gave him further trouble.

Scattered over the extensor surface, chiefly of the fore-arm of this side, you perceive that there are seven more tubercles fairly well developed, with smaller lesions interspersed, whose number it is difficult to determine. These are reddish-brown in color and quite like those we saw upon the forehead. One, over the dorsum of the wrist, exhibits an oblong fissure in its center, covered with a light crust. The lesion is somewhat reddened, and suggests traumatism. The largest of those we find here, is of the size of a bean.

Upon the other forearm, and here also chiefly on the extensor surfaces, we find similar tubercles, eleven in number, the largest of the size of a walnut. They are quite painless to the touch, though not insensitive. On plunging a lancet to the base of one, no fluid escapes; the lesion is evidently a solid new-growth.

Upon the upper and inner face of the right thigh also, we encounter the same developments. Here too is the original tubercle, still existing in precisely the spot where he first observed the earliest symptom of his present disease, now five years ago. There has been evidently some retrograde metamorphosis here, which has, as we noted elsewhere, just stopped short of the grade of ulceration. Where the tubercle is thinned by destruction, you can see a firmly attached scale.

Upon the peroneal borders of the legs, and over the surface of the left thigh, the same lesions are distributed. One, near the left knee, is said to have originated in a "blister," which burst and subsequently discharged a "yellowish stuff" for a long period.

You will note that there is a decided bronze tint of the integument of the legs and feet. It is several shades darker than that of any other portion of the body. The peroneal surfaces of the legs also are somewhat anæsthetic. This anæsthesia, however, is ill-defined, partial, and in no instances limited to outlined areas of the skin.

Our patient complains also of a moderate degree of numbness of the feet, but tells us that he has never had pricking or other abnormal feeling in these parts, such as the "falling asleep" sensation.

(The patient was then removed from the room, at the request of the lecturer.)

Such, gentlemen, is the picture of a case, which, many of you need not be told, is one of tubercular leprosy. Some of you undoubtedly remember the patient affected with the anæsthetic form of the disease presented at this clinic last year; and in the remarks then made, the tubercular form of lepra was briefly described.*

It is somewhat singular that we are enabled to study two cases of this disease, rare in this State, in two successive years. This is the sixth leprous patient of whom I have record in this city. As to the number of those to be found in the United States, our knowledge is yearly increasing. Dr. Rohé, in 1878, estimated the total number of lepers in America at fifty. When I last spoke to you on this subject, I concluded that the number was nearly one hundred.

A record of about 74 cases was obtained by the statistical committee of the American Dermatological Association last year. Since then, Dr. L. F. Saloman has reported 14 cases observed by him in Louisiana; Dr. White, one in Boston; and I have notes of three new cases in Wisconsin and Minnesota, in addition to the three cases previously reported by me from that district, the lepers reported then being still alive. Last month, I saw a

* See a clinical lecture on Anæsthetic Leprosy, *American Practitioner*, Feb. 1879.

Carib Indian affected with the disease, in the amphitheater of the New York hospital. To all these cases we should add the two lepers you have here seen, and a third case, of whose existence I feel very sure, in the person of the little child of the man who has just left the room. She lives in her home in Nebraska, and I have given her name to you, because I think it a matter of importance to endeavor to catalogue every one of these patients known to be living in this country. In other countries, where the disease has made greater progress, the government takes care to keep a register of all cases, but here, where we have a government which is not of the paternal order, the work is left to any man who cares to undertake it, in the interest of science and humanity, and science is always humane. In attempting to eliminate from our scheme of national government all sources of possible evil, we have neglected to endow it with eertain powers which might well be exercised for the benefit of the many.

Now let us say a word as to the diagnosis of this disorder. You will remember that the macular, tubercular and anæsthetic varieties of leprosy are frequently either commingled or merely consecutive stages of one malady. We have just examined a patient exhibiting almost typical features of the tubercular variety, and, as contrasted with the equally typical features of the case of anæsthetic lepra you saw last year, you notice here the absence of a history of hyperæsthesia, the absence of large insensitive atrophic patches, of well defined relatively pigmentless areas, of crusted ulcers profoundly involving the integument, and of anæsthesia distinctly limited to certain tracts of the integument. At the same time, the intimate relationship of the two varieties will be manifest if you notice the similar features of the two cases, which are, in brief, a history of bullæ, much less noticable in the present instance, the ciliary and superciliary alopecia, the inguinal adenopathy, and the well-nigh indescribable something which attests the fact that the two men were alike victims of a constitutional cachexia of grave import.

Here again, observe the polymorphism of the disease which exhibits such a curiously ill-assorted collection of symptoms. This is one of the characteristic signs of syphilis, and we ought to stop for a moment to show that we have not syphilis here.

First, then, we have no history of syphilis in this case, though that will count for but little with us, if we find unmistakable traces of the disease elsewhere. The science of the physician, as Ricord has well said, should always be superior to the assertions of his patient. But the tubercles of syphilis are generally small, often encircled at the base with a collarette of small, fine, dirty scales, beneath which crops out the peculiar mixture of red, brown and yellow discoloration, which medical men have long since agreed to call the "copper color." Then too, syphilitic tubercles could scarcely last for five years without degenerating into ulcers, or becoming covered with the peculiar crusts of the disease. A man, too, who has syphilitic adenopathy of the groins, will rarely show such immense enlargement of the glands as you have just seen, but, should he do this, you would detect, almost certainly, engorgement of the post-cervical or epitrochlear glands, which were not affected in the case of Nanzen. Then, too, adenopathy is a symptom very rarely to be encountered in a case which has lasted for five years. We do not find either, the typical syphilitic ulcer, with its sloughy base and clean-cut edges. The arrangement and locality of the lesions are also quite characteristic, since the tubercular syphilide is apt to appear in groups, and, even when upon the face, never is so conspicuously displayed over the brows as to produce the peculiarly lion-like appearance of the forehead, from which the tubercular variety of leprosy derives one of its many names, leontiasis.

Again, in syphilitic lesions of the throat and larynx, we look rather for the opaline mucous patch in the earlier stages, and, in the later, deeply ulcerative lesions, destroying the submucous tissues or forming membranoid occlusions of the glottis. The tubercles of this throat, considered in connection with the husky voice, recall the signs of leprosy which in the middle ages were thought to be pathognomonic of the disorder.

You will also remember that in vitiligo, there are merely pigmentless areas of integument, without the slightest evidence of the constitutional impairment which is here so conspicuous, while in the disease known as morphœa, there is usually a lilac-tinted border about each infiltrated patch. The bacon-like plâque over the right elbow in Nanzen's case, is commonly seen in the tubercular forms of leprosy ; and I might add that it no way suggests the subcutaneous gummata of syphilis, as the latter are covered with a sound integument, except when in process of degeneration, which results, as a rule, in a typical syphilitic ulcer.

I will not waste your time by pointing out the difference between this disease and the "scrofula," which it was supposed to be. The best use we can make of a blunder of this sort, is to remember that the term scrofula is, more frequently than any other with which I am familiar, made to cover up ignorance, and I urge you to employ it only in those cases where you have the unmistakable evidences of what the best thought of our time has agreed to set apart from all other diseases by that single designation.

The pathological history of lepra is one of new-growth. There is, as you observe in the drawing which I now show you, in all these cases, especially around the thick-walled blood vessels and the cutaneous glands and hair follicles, an abundant development of small, round bodies, closely crowded together, which are masses of living matter, derived from the living epithelia of the normal skin, surrounded by interlacing bundles of connective tissue, not yet undergone the embryonal changes which have been so well described by the later observers. This is true not only of the tubercles, but also of the infiltrations of the surface, such as you saw on the elbow of our patient.

Tschirien has recently observed special changes in leprosy in the form of atrophy of the gray substance of the posterior cornua, and small roundish bodies accumulated in the tissue of the ependyma, with complete integrity of the white substance and the roots of the nerves.*

The questions relating to etiology and contagion, I can dismiss now as before, with a word. The etiology of leprosy is absolutely unknown, and the differences between scientific observers as to contagion, are still unreconciled. My own belief is that the disease is not contagious, and yet I confess that, when it is urged by those who do not accept this view, that the period of incubation is an extremely prolonged one, lasting, it may be, for fifteen or twenty years, we are confronted with an hypothesis,

* *Prog. Méd.*, Mar. 15, 1879, p. 203.

in confutation of which it is well nigh impossible to accumulate proof.

Certainly, if we choose to rely on the statements of our patient, we may conclude that the disease occurs in his family by heredity. There is no proof of the disease in the parental history, and he tells me that he does not know that any of his grandparents, either on the side of father or mother, suffered in a similar way. But we have tolerably distinct evidence of lepra in two of his brothers, and I need not repeat what has been said about his daughter. Heretofore I have stated that no leprous children were known to have been born to leprous parents on American soil, and, if our suspicion regarding this child be well founded, she is the single exception on record. I have made careful inquiries on this point, among the Scandinavian physicians whom I know as having experience of leprosy in the Northwest, and all these gentlemen have denied knowledge of such an occurrence. On the contrary, we know of several cases in which leprous American parents have had children who never presented traces of the parental disorder. It seems to me that the wisest course for us to pursue with regard to the question of heredity, is that described by military men as an "armed neutrality." We can not demonstrate either side of the question, and are compelled to recognize the fact that excellent grounds exist for holding to each view.

As to the prognosis, in the case of our patient, I am inclined to speak much more favorably regarding it, than in the last case brought before you. The anæsthetic patient, Brusher, was a man of dissolute habits and poverty stricken, compelled to seek relief at our public charities. Misery, as the French distinctively term these wretched social conditions, has a great deal to do with other diseases than leprosy. After the date on which you last saw him, Brusher rapidly failed in strength and health. Crops of bullæ continued to appear and burst, and leave sequelæ in atrophied and anæsthetic patches. Emaciation, much darker bronzing of the surface, and finally, chilly and febrile states to these succeeded. In the course of five months, his extreme cachexia contrasted painfully even with the deplorable state in which you first saw him.

I would make simply a less unfavorable prognosis in the case of Nanzen, although we must bear in mind that the tubercular forms of lepra prove fatal more rapidly than the anæsthetic forms. The former period is set, by Professors Bœck and Danielssen, at between eight and nine years. Our present patient is a fairly well-to-do farmer, living in a salubrious part of the country, and able to provide himself with what is really necessary for the preservation of his health. His general appearance is not nearly so unfavorable, apart from the symptoms of his disease, as was that of the poor fellow who limped into this room, nearly one year ago; and yet, curiously enough, the two men had exhibited symptoms for nearly the same length of time.

I prefer to base this favorable opinion upon the considerations named, rather than to lay undue stress upon the supposed value of the external and internal medication, which we shall adopt in the present instance. We shall instruct our patient in the matter of hygiene and food, and give him internally an emulsion of the chaulmoogra oil, which has of late been highly praised in the treatment of lepra. I ought to add, however, that some who have used it, have already pronounced against its efficacy. It is produced by expression from the seeds of the gynocardia odorata, and, according to Dr. Wyndham Cottle, can be given in doses gradually increased from 5 minims to 4 grams daily. It is best administered in emulsion, and, as it has a tendency to constipate the bowels, may require the occasional aid of a cathartic. In the form of an ointment, containing 1 gram of the oil to 4 of lard, it is also applied externally, and we shall also thus employ it in the present case. As a matter of personal experience, I will say that the oil leaves a most persistent taste in the mouth, which is somewhat disagreeable, and which therefore requires correction by some of the devices of elegant pharmacy.

---

On the conclusion of these remarks, a laryngoscopic examination of this patient was made by Dr. H. A. Johnson, Dr. E. F. Ingals, and the lecturer. Dr. Ingals was successful in securing an accurate drawing of the laryngoscopic appearances, which are well represented in the annexed cut. It will be seen that the tubercular development has greatly altered the intralaryngeal appearance, and that the mucous surfaces are also infiltrated and deformed by the same new growth of living matter, which has been observed on the surface of the skin. The tumefied surfaces were reddened, and the mobile parts extremely sluggish in all the

FIG. 1.

LARYNX OF LEPER IN DR. HYDE'S CLINIC.

movements of phonation. Five tolerably distinct tubercles are visible, projecting from the right vocal band, and one also projecting from beneath that on the left, toward its anterior border. Another tubercle is indistinctly shown, projecting from the anterior border of the trachea. In this case, also the larynx exhibits the reddish-yellow color, and the vocal cords the grayish appearance, mentioned by Dr. Elsberg.

The larynges of lepers have rarely been examined. In the *Medical Record*, of New York, for Jan. 4th, 1879, p. 10, will be

FIG. 2.

LARYNX OF LEPER. DR. ELSBERG'S FIRST CASE.

found an interesting report of the throat clinic at the Charity Hospital, New York, service of Prof. Elsberg, whose observations on the larynges of two lepers, are there given. The two patients were in a more advanced stage of the disease than was the man whose case has been given above, but the general direction of the laryngeal changes in all three cases, is remarkably the same. In the accompanying cuts, which have been kindly loaned by Dr. Elsberg to illustrate the report of this lecture, it will be seen that the tumefaction and tubercular studding of the mucous surfaces, advance to deformity from the bulging of the masses which weigh down the mobile parts of the vocal apparatus, and which are accompanied by hypersecretion, while a still more advanced stage can be recognized in greater shapelessness, increased secretion and ulceration.

According to Dr. Elsberg, these changes are preceded by dilatation of the blood vessels in the epiglottis; a peculiar reddish-yellow appearance of the interior of the larynx; gray or dirty discoloration of the vocal bands; and, later, with increased vascularization, tubercles and ulcers.

FIG. 3.

LARYNX OF LEPER. DR. ELSBERG'S SECOND CASE.

It is proper to add that in a letter from Nanzen, received by Dr. Youngstedt, one month after the commencement of treatment by the chaulmoogra oil, the patient writes that he is feeling a great deal better, and that his voice is becoming clearer, while the tubercles are decreasing in size, and the normal sensitiveness returning to the lower extremities. How much of this may be due to the encouragement derived from confidence in his physicians, remains to be determined. But an interesting confirmation of his opinion is furnished in his statement, that his leprous daughter, who is taking the same oil in one minim doses, with external inunction, exhibits an equally marked improvement, the tubercles diminishing in size upon her extremities.

## Figures and Tables

**FIG. 1. f1:**
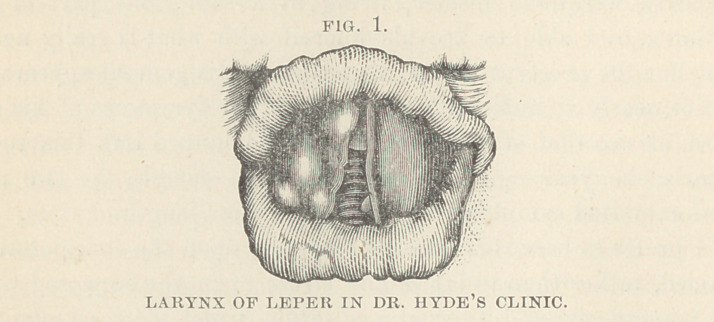


**FIG. 2. f2:**
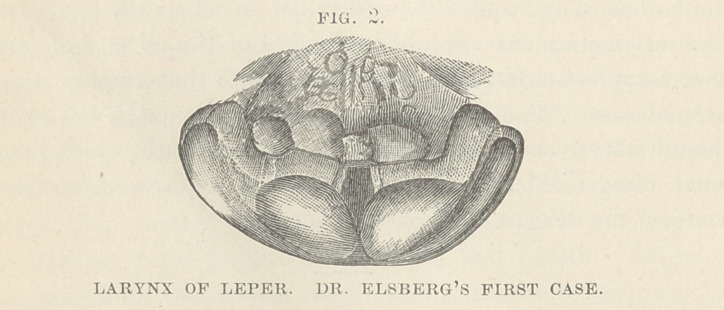


**FIG. 3. f3:**